# A Puerto Rican Variant of Lady Windermere Syndrome: Reanalyzing the Characteristics of Non-tuberculous Mycobacterial Infection

**DOI:** 10.7759/cureus.63900

**Published:** 2024-07-05

**Authors:** Tyffany Sebastian Hurtado, Amanda Alvelo, Gabriel Colon Estarellas, Sharon Velez Maymi

**Affiliations:** 1 Internal Medicine, San Juan City Hospital, San Juan, PRI

**Keywords:** mycobacterium avium complex (mac), tree-in-bud, hispanic woman, nontuberculous mycobacterium (ntm), lady windermere syndrome

## Abstract

Lady Windermere syndrome (LWS) is a disease caused by a non-tuberculous *Mycobacterium* (NTM) that is commonly found in thin women who voluntarily suppress their cough reflex. The NTM that causes this syndrome is *Mycobacterium avium* complex, an organism commonly present in chlorinated city water and soil. Patients with LWS are tall, lean, elderly white women. We report a case of an immunocompetent 81-year-old thin Puerto Rican female with a recurrent cough since childhood, who was misdiagnosed with tuberculosis (TB) and prophylactically treated. While the patient fitted the clinical picture of NTM pulmonary infection based on symptoms, imaging, and microbiologic findings, her demography and morphologic features were not completely consistent with published findings. The incidence and prevalence of NTM lung disease are rising worldwide due to the aging population, increased use of immunosuppressive medications, and prevalence of chronic pulmonary obstructive disease and bronchiectasis. The goal of this report is to increase awareness of LWS as one of the diagnoses that should be considered in patients presenting with clinical findings resembling TB and bring attention to the different clinical characteristics this patient with LWS possessed.

## Introduction

Lady Windermere syndrome (LWS) is a non-tuberculous *Mycobacterium* (NTM) lung condition, commonly caused by *Mycobacterium avium* complex (MAC), an organism commonly present in chlorinated city water and soil. This syndrome was first described by Reich and Johnson [1] in 1992. Reich and Johnson reported six cases of elderly female patients where the right middle lobe of the lung was affected. The syndrome’s name stems from Oscar Wilde’s Play “Lady Windermere’s Fan” which uses comedy and satire to illustrate the polite manners of the Victorian Era [2]. Patients with this syndrome voluntarily suppress their cough, which has been proposed to play a role in developing nodular bronchiectasis associated with NTM lung infection from retained mucus [3]. A prospective study by Kim et al. identified patients with NTM lung disease to be tall, lean, white elderly women, with associated scoliosis, pectus excavatum, or mitral valve prolapse [4]. We report the case of an 81-year-old female with LWS secondary to MAC of short stature and non-Caucasian descent. This clinical case was presented in the 2023 ACP PR Chapter Clinical Vignette Competition on November 18, 2023.

## Case presentation

The patient was an 81-year-old female with a history of bronchiectasis, chronic obstructive pulmonary disease, allergic rhinitis, hypertension, and hypercholesterolemia. The patient was a lifetime nonsmoker but had been previously exposed to secondary smoke. The patient was being followed outpatient by a pneumologist due to an intermittent productive cough. A chest CT scan was performed and showed extensive bronchiectatic changes involving both lungs with nodular opacities in a tree-in-bud configuration more prominent in the right middle lobe and lingula. The CT was followed by a positron emission tomography (PET) scan that was remarkable for bilateral hypermetabolic lobar infiltrates and nodular opacities more prominent in the right middle lobe and lingula, for which an active infectious granulomatous disease versus a superimposed neoplastic process could not be excluded. Left lung biopsy with acid-fast Ziehl-Neelsen stain was positive for acid-fast bacilli (AFB). Based on these findings, the patient was started on RIPE (rifampin, isoniazid, pyrazinamide, ethambutol) therapy for suspected tuberculosis (TB) two weeks before being admitted to our institution.

**Figure 1:**
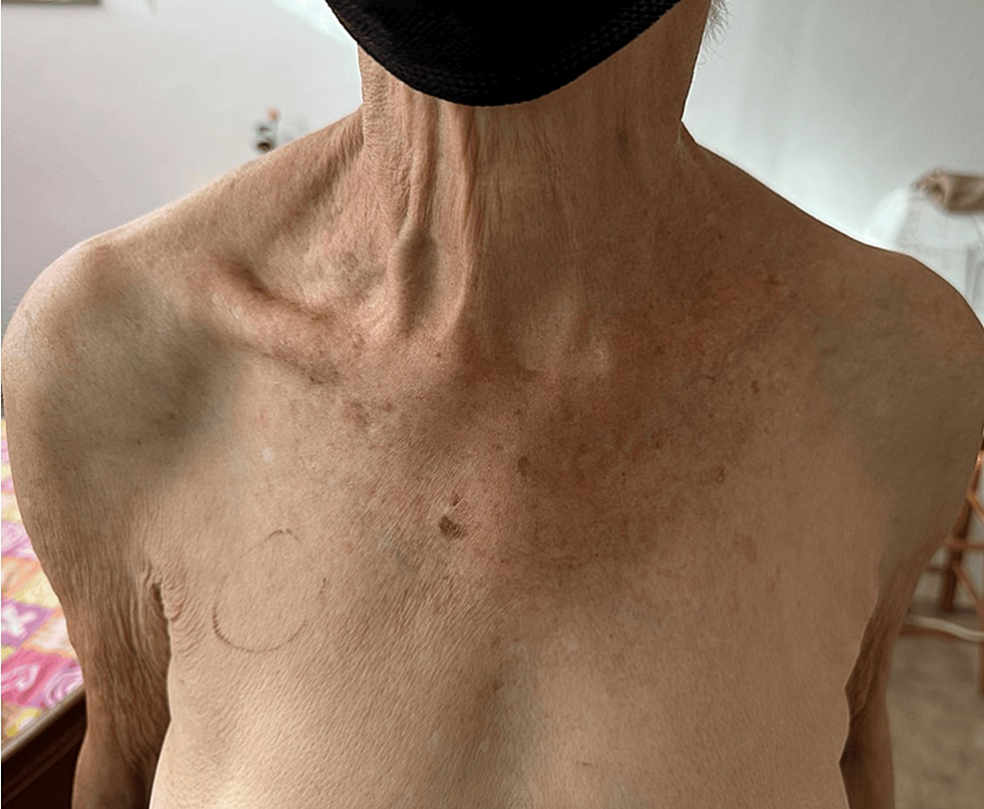
View of pectus excavatum present in the patient.

The patient presented to our institution with generalized abdominal pain of two days of evolution, associated with general malaise, nausea, emesis, anorexia, and sensation of abdominal fullness. The patient denied fever, chills, night sweats, hemoptysis, unintentional weight loss, recent travel, immunocompromised state, changes in bowel habits, or a history of incarceration. On physical examination, the patient was noted to have a petite frame with a height of 4 ft 3 in (129.54 cm) and a weight of 81 pounds. She was awake; alert; oriented to time, place, and person; cooperative with examination; polite; and in no apparent distress. Abdominal examination revealed bowel sounds and tenderness on deep palpation more prominent on the right side. There was no guarding, rebound tenderness, or organomegaly. In addition to her chief complaint of abdominal pain, the patient also complained of a chronic “nagging” cough. Despite the patient complaining of a chronic cough, no coughing spells were witnessed during the examination. Respiratory examination revealed bilateral decreased breath sounds and no wheezes or crackles. The chest was remarkable for pectus excavatum (Figure 1). Her politeness, along with never coughing in the presence of medical staff, despite her complaining of it, are characteristics of patients with LWS.

**Figure 2:**
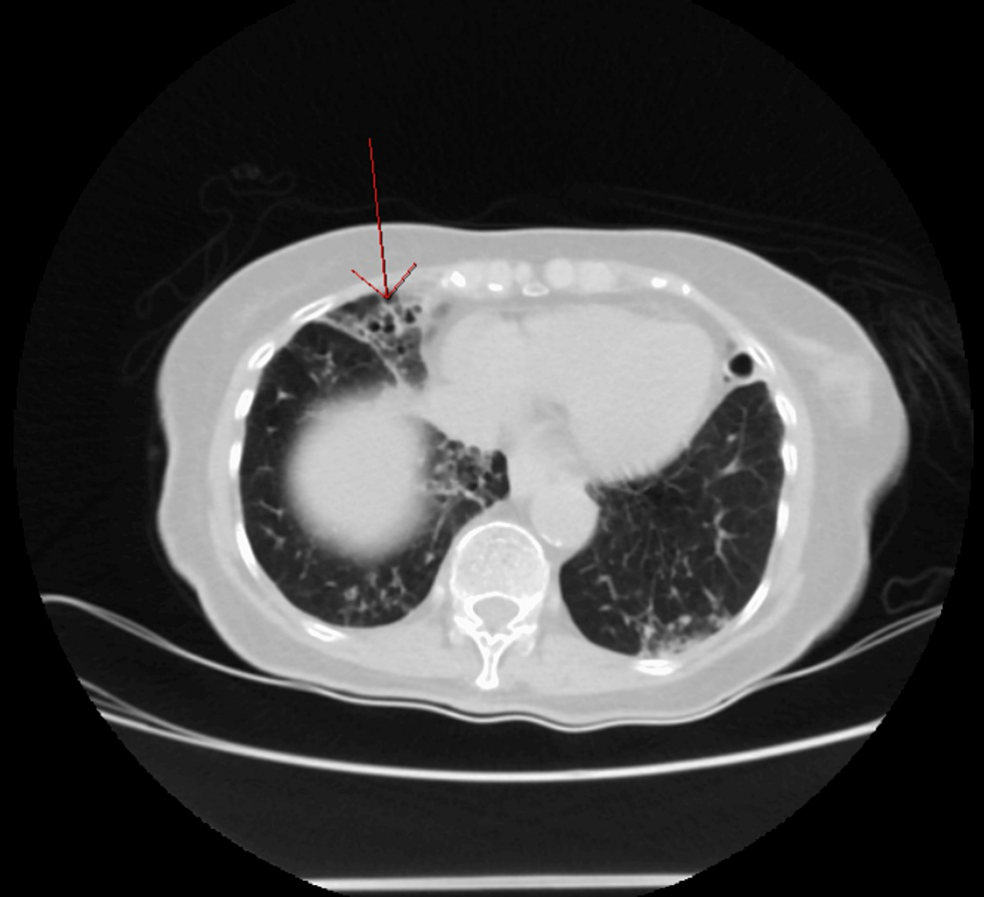
Nodular bronchiectasis involving the right middle lobe in the axial cut of CT abdomen/pelvis with intravenous contrast.

**Figure 3:**
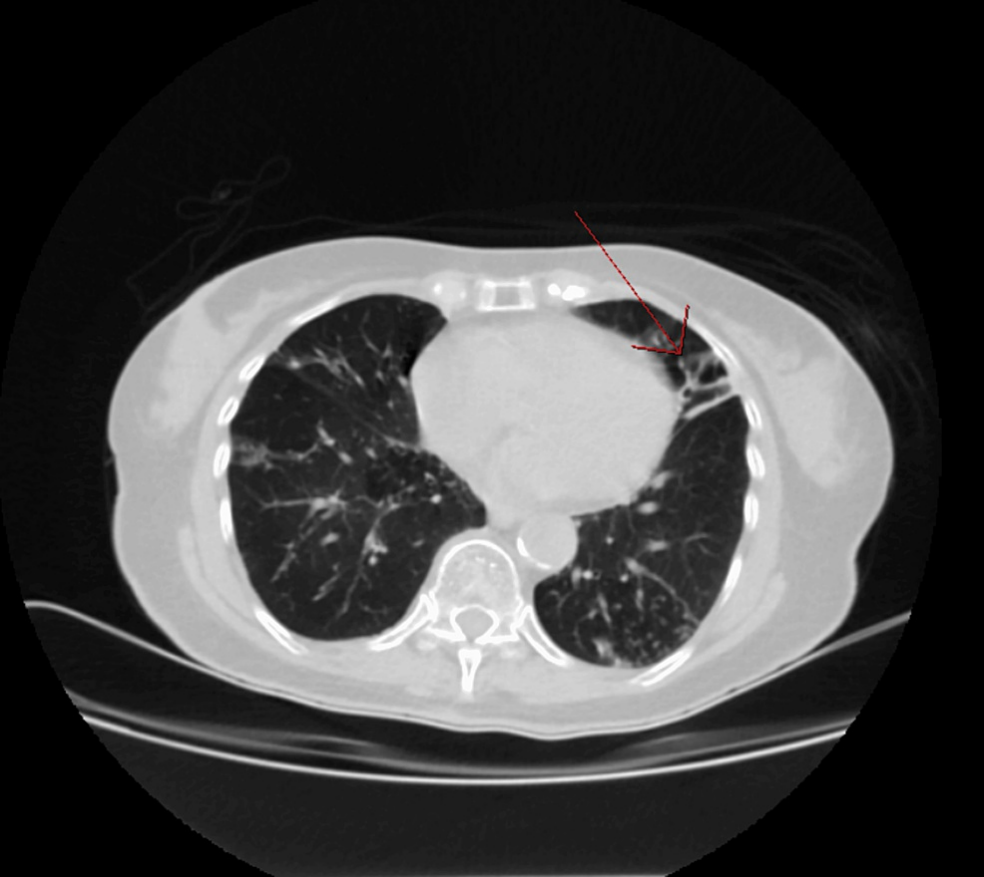
Nodular bronchiectasis involving the lingula of the left lung in CT abdomen/pelvis with intravenous contrast.

**Figure 4:**
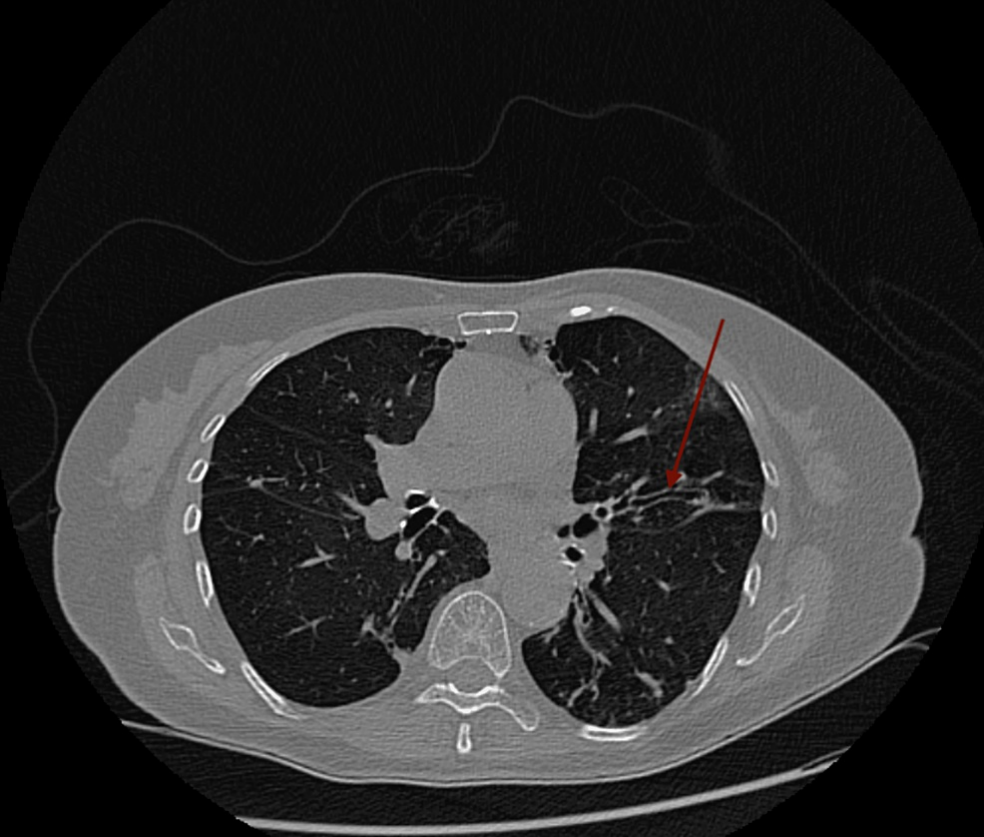
Cylindrical bronchiectasis in CT thorax with oral contrast.

Laboratory results were as follows: white blood cell, 3.9 × 10^3^/µL (4.8-10.8 × 10^3^/µL); hemoglobin, 12 g/dL (12.0-14 g/dL); hematocrit, 35% (37-52%); platelet, 238 × 10^3^/µL (150-450 × 10^3^/µL), with a differential of neutrophils, 56% (42.20-75.20%); monocytes, 19% (1.70-9.30%); lymphocytes, 23% (20.50-51.10%); and eosinophils, 0.50% (0-10%). The complete metabolic panel was remarkable for transaminitis with aspartate transaminase of 1,509 IU/L, alanine transaminase of 707 IU/L, and total bilirubin of 2.63 mg/dL (0.40-1.30 mg/dL). Amylase was 100 U/L and lipase was 57 U/L. The patient was negative for hepatitis A, B, and C. Given the transaminitis, hepatotoxic RIPE therapy was placed on hold. Abdominal/pelvic CT imaging was ordered and was unremarkable for liver enlargement or lesions. However, it was remarkable for bilateral bronchiectasis involving the right middle lobe and lingula (Figures 2, 3). Based on these findings, a CT thorax was ordered, which revealed cylindrical bronchiectasis (Figure 4). Infectious disease service was consulted due to the outpatient diagnosis of TB on RIPE therapy.

The AFB stain smear of sputum was positive twice, but the *Mycobacterium tuberculosis* complex was not detected by GeneXpert; hence, TB was ruled out. The final mycobacteria culture was remarkable for MAC by a DNA probe. Based on the clinical, radiographic, and microbiological criteria established by the Infectious Diseases Society of America and the American Thoracic Society (IDSA/ATS) for the diagnosis of NTM pulmonary disease, the RIPE therapy was discontinued. Transaminitis secondary to RIPE therapy resolved and the patient was discharged home. Upon discharge, the patient followed up with an infectious disease specialist for the treatment of NTM pulmonary infection.

## Discussion

We report a case of LWS caused by an NTM infection that presented without the common phenotype and race that has been associated with this syndrome. This is relevant in clinical practice as there is an increase in the prevalence of NTM lung disease worldwide. In the United States, from 2008 to 2015, the annual prevalence increased from 6.8 to 11.7 per 100,000 persons [5]. In South Korea, the prevalence increased from 6.7 cases to 39.6 cases per 100,000 persons from 2007 to 2016 [6]. More clinicians are being presented with NTM infections and correct diagnosis is paramount for the successful treatment of this uncommon pathology, which can easily be mistaken for TB, as was the case with this patient.

The patient fulfilled the unique characteristics of LWS, as she was a thin, well-mannered, older woman who suppressed her cough reflex out of choice with imaging remarkable for cylindrical bronchiectasis and nodules in the right middle lobe and lingula. Similar radiographic findings were observed by Kim et al. [4]. The study also found scoliosis, mitral valve prolapse, pectus excavatum, and an average height of 164.7 cm to be features associated with NTM disease. The patient in this case only possessed pectus excavatum. Her height was 149.8 cm, which was below the average reported by Kim et al.. Another study by Kartalija et al. that compared morphotypes of individuals with NTM disease and compared with control subjects reported the average height of cases with NTM disease to be 166.94 cm [7]. Regarding demographics, Kim et al. found 95.2% of cases with NTM lung disease to be females and 90.5% to be white. Kartalija et al. found 85% to be females and 93% to be white. In this case, the patient was Puerto Rican.

The treatment of NTM caused by MAC depends on the presence of cavitary bronchiectasis and the severity of the disease. Patients without cavitary bronchiectasis, which is mild to moderate in severity, are treated with azithromycin, rifampin, and ethambutol three times per week [8]. Patients who are taking other medications that interact with rifampin or have had previous hepatoxicity due to rifampin can be prescribed rifabutin instead. Patients with cavitary bronchiectasis or severe nodular bronchiectasis are treated with azithromycin, rifampin, and ethambutol daily plus parenteral amikacin. Amikacin is given three times per week for two to three months. The treatment is continued until sputum cultures are consecutively negative for at least 12 months [8]. The duration of treatment can range between 15 and 18 months. Response to treatment is assessed by obtaining sputum cultures every two to three months and repeating imaging every six months. Patients who fail treatment or are unable to tolerate drugs can be managed surgically.

## Conclusions

We describe a patient with LWS and NTM infection. While the patient met the criteria established by IDSA/ATS for the diagnosis of NTM, she did not completely fit the morphological and demographic prototype of this condition based on previous literature. Our patient was neither tall nor white. Our patient was a petite woman with a height of 149.8 cm and Hispanic. This case highlights the morphological and demographic variations that can be present in a patient with LWS and NTM lung disease in Puerto Rico. This is important to consider to provide the best care and pharmacological treatment and avoid iatrogenic harm. This was the case in our patient who developed transaminitis from hepatotoxic medications she was receiving to treat TB.

## References

[1] Reich JM, Johnson RE (1992). Mycobacterium avium complex pulmonary disease presenting as an isolated lingular or middle lobe pattern. The Lady Windermere syndrome. Chest.

[2] Donatelli C, Mehta AC (2015). Lady Windermere syndrome: Mycobacterium of sophistication. Cleve Clin J Med.

[3] Kumfer AM Edriss H (2017). Lady Windermere syndrome. Southwest Respir Crit Care Chron.

[4] Kim RD, Greenberg DE, Ehrmantraut ME (2008). Pulmonary nontuberculous mycobacterial disease: prospective study of a distinct preexisting syndrome. Am J Respir Crit Care Med.

[5] Winthrop KL, Marras TK, Adjemian J, Zhang H, Wang P, Zhang Q (2020). Incidence and prevalence of nontuberculous mycobacterial lung disease in a large U.S. managed care health plan, 2008-2015. Ann Am Thorac Soc.

[6] Lee H, Myung W, Koh WJ, Moon SM, Jhun BW (2019). Epidemiology of nontuberculous mycobacterial infection, South Korea, 2007-2016. Emerg Infect Dis.

[7] Kartalija M, Ovrutsky AR, Bryan CL (2013). Patients with nontuberculous mycobacterial lung disease exhibit unique body and immune phenotypes. Am J Respir Crit Care Med.

[8] Daley CL, Iaccarino JM, Lange C (2020). Treatment of nontuberculous mycobacterial pulmonary disease: an Official ATS/ERS/ESCMID/IDSA clinical practice guideline. Clin Infect Dis.

